# Surgeons’ Interactions With and Attitudes Toward E-Patients: Questionnaire Study in Germany and Oman

**DOI:** 10.2196/14646

**Published:** 2020-03-09

**Authors:** Ken Masters, Teresa Loda, Jonas Johannink, Rashid Al-Abri, Anne Herrmann-Werner

**Affiliations:** 1 Medical Education and Informatics Department College of Medicine and Health Sciences Sultan Qaboos University Al-Khoud Oman; 2 Medical Department VI Psychosomatic Medicine and Psychotherapy University Hospital Tübingen Tübingen Germany; 3 Department of Surgery and Transplantation University Hospital Tübingen Tübingen Germany

**Keywords:** internet, e-patient, internet-informed patient, doctor-patient relationship, attitude, digital health, technology, e-physician, empowerment, physician, communication

## Abstract

**Background:**

Doctors’ interactions with and attitudes toward e-patients have an overall impact on health care delivery.

**Objective:**

This study aimed to gauge surgeons’ interactions with e-patients, their attitudes toward those e-patient activities, the possible impact on the delivery of health care, and the reasons behind those activities and attitudes.

**Methods:**

We created a paper-based and electronic survey form based on pertinent variables identified in the literature, and from March 2018 to July 2018 we surveyed 49 surgeons in Germany and 59 surgeons in Oman, asking them about their interactions with and attitudes toward e-patients. Data were stored in Microsoft Excel and SPSS, and descriptive statistics, Pearson correlations, and chi-square tests were performed on the data.

**Results:**

Of our sample, 71% (35/49) of the German surgeons and 56% (33/59) of the Omani surgeons communicated electronically with their patients. Although the German surgeons spent a greater percentage of Internet usage time on work-related activities (χ^2^_18_=32.5; *P*=.02) than the Omani surgeons, there were many similarities in their activities. An outstanding difference was that the German surgeons used email with their patients more than the Omani surgeons (χ^2^_1_=9.0; *P*=.003), and the Omani surgeons used social media, specifically WhatsApp, more than the German surgeons (χ^2^_1_=18.6; *P*<.001). Overall, the surgeons were equally positive about the most common e-patient activities such as bringing material from the internet to the consultation (mean 4.11, SD 1.6), although the German surgeons (mean 3.43, SD 1.9) were more concerned (*P*=.001) than the Omani surgeons (mean 2.32, SD 1.3) about the potential loss of control and time consumption (German: mean 5.10, SD 1.4 and Omani: mean 3.92, SD 1.6; *P*<.001).

**Conclusions:**

The interactions show a high degree of engagement with e-patients. The differences between the German and the Omani surgeons in the preferred methods of communication are possibly closely linked to cultural differences and recent historical events. These differences may, moreover, indicate e-patients’ desired method of electronic communication to include social media. The low impact of surgeons’ attitudes on the activities may also result from a normalization of many e-patient activities, irrespective of the doctors’ attitudes and influences.

## Introduction

### Background and Literature

The overall impact of the engaged and better-informed patient on the patient-doctor relationship can be positive or negative [1,2]. The internet, moreover, has introduced a new dimension to the engaged and better-informed patient (ie, the e-patient). In short, e-patients are patients who are “equipped, enabled, empowered and engaged in their health and health care decisions” [3] and who use “the Internet to gather information about a medical condition of particular interest to them” [3].

This is not the place to give a more detailed account of e-patient activities; nevertheless, it is useful to note that typical activities involve searching for medical and health-related information on the internet, joining Web-based patient discussion groups, communicating electronically with their physicians, accessing their electronic medical records, accessing their laboratory results, using personal health records, researching their physician’s electronic footprint, and other electronically based health-related activities [4-16].

The term *e-patient* has only recently gained traction in the medical literature. A PubMed/ Medical Literature Analysis and Retrieval System Online search by the authors for articles published before 2011 and referring to the term *e-patient* or *epatient* in the title or abstract revealed only 5 citations. The same search for the years 2011 to 2019 conducted in March 2019 revealed 24 citations.

Similar to the impact of the engaged patient before the advent of the internet, the e-patient’s activities’ impact on the patient-doctor relationship can vary, with some doctors reporting a worsening relationship and others reporting a strengthening of the relationship and improved health care [11,17-19].

In general, a more engaged patient can result in better health outcomes and lower costs [20,21]. In the domain of surgery, more informed patients have experienced less postoperative pain than uninformed patients [22]. It is known that e-patients consult the internet pre surgery and post surgery, and approximately 20% of e-patients consult websites recommended by their surgeon or doctor and rate those websites higher in quality than other sites they have consulted [12]. It is also known that e-patients use the internet to research their surgeons, although this is not always welcomed by surgeons [12].

There is strong evidence that doctors’ attitudes toward patients’ use of the internet, especially if they recommend sites to their patients, directly influence those patients’ usage; the quality of material found; and, by extension, the patient-doctor relationship [12]. Worryingly, however, international research shows that approximately 65% to 80% of e-patients *do not* share this information with their doctors, primarily because of their doctors’ negative attitudes toward the internet [19]. One can only speculate the harm to the patient-doctor relationship and the communication breakdown that could result from this.

### Setting

In Germany and Oman, approximately 96% and 80% of the population has access to the internet, respectively [23,24]. A 2009 survey indicated that there were some 40 million e-patients in Germany [25]; based on internet usage in Germany from 2016, this figure can be calculated at approximately 49 million or higher [26]. This number represents a potentially great impact on the patient-doctor relationship.

There are currently no similar figures for e-patients in Oman. That said, the internet usage adoption rate in Oman, although behind Germany, has followed the trend of increasing usage seen in Germany and other countries [4]. Given this, there is reason to believe that e-patient activities will also follow similar trends. There is, however, always the possibility of differences in practice because of differences in culture, history, and other influencing factors, so one should not too easily make assumptions about one country’s practices based on practices in others.

### Aim of the Study

This study focuses on surgeons’ attitudes toward e-patients in Germany and Oman. The aim of this study was to gauge surgeons’ interactions with e-patients, their attitudes toward those e-patient activities, the possible impact on the delivery of health care, and the reasons behind those activities and attitudes. This knowledge would give us some idea of the impact of e-patient activity on the patient-surgeon relationship in these 2 countries.

## Methods

### Variables

As part of the study was a comparison between the 2 countries, the first independent variable was the country in which the surgeon resided. Our selection of further independent variables to be studied was guided by the literature that had indicated possible predictors of attitudes toward e-patients. These included the doctors’ age and gender, amount of internet usage, and work-related time on the internet and the assumption that patients with chronic conditions who have access to the internet are more likely to be engaged in self-care and communicate electronically with doctors [2,9,13,16].

In addition, as there would be variations in usage and we wished to know if these variations might have an impact on attitudes, we asked questions about the specific internet sites that doctors visited.

The dependent variables were guided by the knowledge and activities described as typical e-patient activities, as listed above, also derived from the literature on the e-patient [4-16]. The last variables on attitudes toward the e-patient were questions derived from a study by Moick and Terlutter [27], described in more detail below.

### Questionnaire

The description of the questionnaire design and delivery follows the Checklist for Reporting Results of Internet E-Surveys checklist (see Multimedia Appendix 1).

In Germany, the questionnaire was delivered to all surgeons from the General, Transplant, Visceral, Heart, and Orthopedic and Trauma Surgery Departments from the University Hospital, Tübingen, and the affiliated *Berufsgenossenschaftliche Unfallklinik*, Tübingen. The questionnaire was on paper and in an electronic format, using Google Forms. English fluency could be assumed among German doctors working in an academic environment, so the questionnaire was administered in English. Ethics approval was obtained from the University of Tübingen’s Medical Ethics Committee (No. 001/2018BO2).

In Oman, the questionnaire was delivered to surgeons in the Department of Surgery at Sultan Qaboos University Hospital (including those surgeons affiliated to the department from the Oman Medical Association). The questionnaire was on paper and in an electronic format, using SurveyMonkey (SVMK Inc, San Mateo, CA). As all doctors in Oman need to be fluent in English, the questionnaire was delivered in English. Ethics approval was obtained from the Sultan Qaboos University College of Medicine Research Ethics Committee (MREC#1628).

In all cases, the information sheet and consent form contained the title and a brief description of the research project, names and contact details of the researchers, a brief statement about risks to the participants, confidentiality, storage of information (256-bit encryption), the voluntary nature of the participation, and permission to retain (or obtain) a copy of the informed consent form. All surgeons signed the informed consent form or checked an appropriate box on the electronic form. After the collection of the paper forms, the signed consent form was separated from the questionnaire and stored in a separate location.

For the core of the questionnaire design, we elected to use the relevant part of the survey form designed by Moick and Terlutter [27]. Moick and Terlutter’s questionnaire was based on issues raised in the medical literature, and they determined it to be internally consistent. The questionnaire consists of 6 items about attitudes of online informed patients, ranging from 1 (*absolutely disagree*) to 7 (*absolutely agree*). As a double check, we inspected the literature that Moick and Terlutter had cited in the construction of their questionnaire. We did this to ensure that those sources did support the construction of their questions and accepted the questions as valid.

In addition to the questions from Moick and Terlutter, other literature [13] and surgeons were consulted to add further questions. The final version of the questionnaire is provided in Multimedia Appendix 2. The Web-based version was delivered on a single, scrollable screen so that the surgeons could review all their answers before submission.

### Questionnaire Delivery

The surgeons were contacted through internal electronic mailing lists and WhatsApp groups and directed to the Web-based forms through URLs. Where surgeons preferred the paper form, these were delivered to them. No incentives were offered to the surgeons for the completion of the form. The data collection was performed from March 2018 to July 2018.

To maintain confidentiality and on the grounds that these are extremely easy to circumvent, no checks or preventative measures through cookies or internet protocol address identification were taken.

### Data Analysis

All questionnaires were analyzed. Data were stored in Microsoft Excel 2016 and SPSS (version 25). Means, standard deviations, and frequencies were calculated. The data were normally distributed. Owing to this, Pearson correlations were run to examine correlations between the appropriate variables described above. To test for the differences between the German and Omani surgeons, chi-square tests and *t* tests for independent samples were conducted. Corrections for multiple testing were based on Bonferroni correction. (For the sake of brevity, the Results section speaks of the *German* and the *Omani* surgeons, although the reader should remember that this does not refer to their nationality but their location at the time of this study.)

## Results

### General

A total of 38% (49/128) German surgeons and 71% (59/83) Omani surgeons completed the survey. Of all the surgeons, only 6 Omani surgeons completed the survey on paper, so the number was too small to run any comparative statistical tests. In addition, the tables below show summary data only. Multimedia Appendix 3 contains charts with more details.

### Demographics and Setting

We established the surgeons’ age; gender; and whether the majority of their patients suffered from chronic, acute, or roughly the same types of condition. Table 1 summarizes these results.

**Table 1:** Surgeons’ age, gender, and majority of patients, by country.

Category		Germany (n=49)	Oman (n=59)	Overall (n=108)		Statistics				
						*t* value (*df*)		Chi-square value (*df*)		*P* value
**Age (years)**					4.43 (104)		N/A^a^		.001	
	Mean (SD)	36.27 (8.83)	44.19 (9.59)	40.56 (10.07)						
	Range	26-62	25-61	25-62						
**Gender, n (%)**					N/A		2.2 (2)		.33	
	Female	17 (34.7)	13 (22.0)	30 (27.8)						
	Male	31 (63.3)	45 (76.3)	76 (70.4)						
	Unknown gender	1 (2.0)	1 (1.7)	2 (1.9)						
**Types of condition, n (%)**					N/A		10.5 (2)		.01	
	Chronic	5 (10.2)	22 (37.3)	27 (25.0)						
	Acute	10 (20.4)	8 (13.6)	18 (16.7)						
	Both	34 (69.4)	29 (49.2)	63 (58.3)						

^a^Not applicable.

For most of the practices and attitudes given below, the figures will be viewed in light of the figures in Table 1.

### Internet Usage

We measured the surgeon’s internet usage, both broadly and more specifically, their knowledge and usage of sites and apps. When regarding the hours per day spent on the internet, there was no significant effect for origin (*P=*.67), age (*P=*.06), gender (*P=*.97), or condition type (*P=*.67). Table 2 shows the number of hours spent per day on the internet.

The German surgeons had a higher percentage of time (mean 60.04, SD 17.95) devoted to work-related activities (*t*_106_=−3.72; *P*<.001) than the Omani surgeons (mean 45.34, SD 23.10). There was no difference between the work-related internet time and age (*P=*.15), gender (*P=*.21) or condition type (*P=*.91).

Delving further into work-related activities, we asked which sites and apps the surgeons knew about and used at least once per month. This would help to complete an overall picture of the surgeons’ general familiarity with medically related websites and apps. (As can be seen from the questionnaire in Multimedia Appendix 1, examples of each of these categories were provided in case the subjects were not sure of what was meant by the category.) Tables 3 to 6 provide these figures.

Although there is similar knowledge of general references, databases, and journals, a significantly higher proportion of the German surgeons have knowledge about books, videos, networking sites, official sites, and magazines (Table 3).

Interestingly, however, when looking at the *usage* of these sites, most of these differences are reduced or even disappear. The use of databases is an exception, with usage by Omani surgeons far less than that by German surgeons (Table 4).

When viewing surgeon’s knowledge of apps, again we see differences, with the German surgeons usually having greater knowledge than the Omani surgeons. A notable exception is Continuing Professional Development (CPD) apps (Table 5).

With app usage, most of the differences (including CPD) are removed, except for tools apps (Table 6).

**Table 2:** Hours spent per day on the internet.

Country	Hours						
	0	1-2	3-4	5-6	7-8	9-10	>10
Germany, n (%)	0 (0)	21 (42.9)	15 (30.6)	5 (10.2)	3 (6.1)	2 (4.1)	3 (6.1)
Oman, n (%)	0 (0)	23 (39.0)	24 (40.7)	9 (15.3)	2 (3.4)	1 (1.7)	0 (0)
Overall, n (%)	0 (0)	44 (40.7)	39 (36.1)	14 (13.0)	5 (4.6)	3 (2.8)	3 (2.8)

**Table 3:** Surgeons’ knowledge of sites, by country.

Site	Germany (n=49), n (%)	Oman (n=59), n (%)	Total (N=108), n (%)	Statistics	
				Chi-square value (*df*)	*P* value
Books	46 (93.9)	42 (71.2)	88 (81.5)	8.4 (1)	.004
Videos	36 (73.5)	27 (45.8)	63 (58.3)	8.0 (1)	.005
General references	41 (83.7)	48 (81.4)	89 (82.4)	0.0 (1)	.90
Networking sites	22 (44.9)	10 (16.9)	32 (29.6)	9.7 (1)	.002
Official/institutional	32 (65.3)	24 (40.7)	56 (51.9)	6.1 (1)	.01
Databases	49 (100.0)	55 (93.2)	104 (96.3)	2.6 (1)	.11
Journals	44 (89.8)	50 (84.7)	94 (87.0)	0.3 (1)	.57
Magazines	36 (73.5)	11 (18.6)	47 (43.5)	32.0 (1)	.001

**Table 4:** Surgeons’ use of sites at least once per month, by country.

Site	Germany (n=49), n (%)	Oman (n=59), n (%)	Total (N=108), n (%)	Statistics	
				Chi-square value (*df*)	*P* value
Books	33 (67.3)	29 (49.2)	62 (57.4)	2.9 (1)	.09
Videos	24 (49.0)	17 (28.8)	41 (38.0)	4.1 (1)	.04
General references	31 (63.3)	39 (66.1)	70 (64.8)	0.3 (1)	.58
Networking sites	9 (18.4)	4 (6.8)	13 (12.0)	3.2 (1)	.08
Official/institutional	10 (20.4)	15 (25.4)	25 (23.1)	0.5 (1)	.48
Databases	48 (98.0)	42 (71.2)	90 (83.3)	12.1 (1)	.001
Journals	31 (63.3)	36 (61.0)	67 (62.0)	0.0 (1)	.99
Magazines	9 (18.4)	7 (11.9)	16 (14.8)	0.7 (1)	.40

**Table 5:** Surgeons’ knowledge of these apps, by country.

App Types	Germany (n=49), n (%)	Oman (n=59), n (%)	Total (N=108), n (%)	Statistics	
				Chi-square value (*df*)	*P* value
Monitoring	25 (51.0)	14 (23.7)	39 (36.1)	8.3 (1)	.004
Information	34 (69.4)	35 (59.3)	69 (63.9)	1.0 (1)	.33
Continuing Professional Development	20 (40.8)	36 (61.0)	56 (51.9)	4.8 (1)	.03
Tools	34 (69.4)	22 (37.3)	56 (51.9)	10.5 (1)	<.001
Videos	27 (55.1)	32 (54.2)	59 (54.6)	0.00 (1)	.99

**Table 6:** Surgeons’ use of these apps at least once per month, by country.

App Types	Germany (n=49), n (%)	Oman (n=59), n (%)	Total (N=108), n (%)	Statistics	
				Chi-square value (*df*)	*P* value
Monitoring	8 (16.3)	5 (8.5)	13 (12.0)	1.5 (1)	.22
Information	27 (55.1)	26 (44.1)	53 (49.1)	1.1 (1)	.29
Continuing Professional Development	13 (26.5)	24 (40.7)	37 (34.3)	2.6 (1)	.12
Tools	23 (46.9)	15 (25.4)	38 (35.2)	5.2 (1)	.02
Videos	16 (32.7)	29 (49.2)	45 (41.7)	3.3 (1)	.07

### E-Patient

We wanted to know if the surgeons engaged in the types of communication with e-patients that the literature had identified, their experience with e-patients, and their attitudes toward some of the implications of e-patient activities. The last set of questions would also allow a comparison with the data from the study by Moick and Terlutter [27].

We began our investigation of the surgeons’ interactions and attitudes toward the e-patient by examining whether or not the surgeons were aware of the terminology. Of these surgeons, fewer German (23/49, 46%) than Omani (34/59, 57%) surgeons had heard of the term *e-patient*; however, this difference is not significant (χ^2^_1_=0.6; *P=*.23).

Of these surgeons, 71% (35/49) German and 55% (33/59) Omani surgeons communicated electronically with patients (χ^2^_1_=2.8; *P=*.10).

Given that electronic communication can take various forms, we wanted to see if there were differences in the methods of electronic communication between the 2 countries. Table 7 shows the method of communication used by the surgeons who communicate electronically with patients.

The figures for email communication, WhatsApp, and Twitter are significantly different, with the German doctors preferring email, and the Omani surgeons use both email and WhatsApp equally, and some Omani surgeons use Twitter. We should also note that there are several *Messenger* apps (eg, WhatsApp Messenger and Facebook Messenger), so the 2 items from that category might actually belong elsewhere, for example, WhatsApp or Facebook. Either way, this is a social media site, rather than email. These differences in methods of electronic communication are discussed in more detail in the Discussion section.

In addition to knowing how many surgeons communicate electronically with their patients, we wanted to know what percentage of their patients use email to communicate with them.

The results indicate that the German surgeons communicated with a larger percentage of their patients via email than the Omani surgeons (χ^2^_9_=25.1; *P=*.003). In addition, when looking on a metric level, for the German surgeons, email usage was strongly associated with their overall amount of internet usage (Pearson *r*=0.522; *P*<.001); this association was not found with the Omani surgeons.

Similarly, we wished to find out what percentage of their patients communicate with the surgeons via social media. The Omani surgeons communicated with a far greater amount of their patients via social media than the German surgeons (χ^2^_11_=48.6; *P<*.001).

An important aspect of the e-patient is the patient who brings material from the internet to the doctor. Of the surgeons, 89% (44/49) German and 84% (50/59) Omani surgeons indicated that patients bring information from the internet to the consultation (χ^2^_12_=18.9; *P=*.09).

Correspondingly, we wished to know how frequently surgeons recommend websites or apps to their patients. Of the surgeons, 40% (20/49) German and 49% (29/59) Omani surgeons recommended a website or app to their patients at least once per month (χ^2^_1_=3.5; *P=*.06).

For the German surgeons, the number of website recommendations was strongly associated with the amount of internet usage (Pearson *r*=0.326; *P*=.02).

Interestingly, the impact of these recommendations on the patients appeared minimal, as there was no association between the number of recommendations and the frequency that patients are bringing information from the internet to the consultation (*P=*.06).

Of the specific websites or apps recommended by the German surgeons, 19 sites were mentioned, but no site was recommended more than once. From the Omani surgeons, 13 sites were mentioned. The 3 most recommended sites were Google (11), Medscape, and YouTube (3 each). All other sites were recommended only once. (Of interest, the top 3 Omani sites [Google, Medscape, and YouTube] were not recommended at all by the German surgeons.)

**Table 7:** Surgeons’ methods of electronic communication with patients, by country.

Method		Germany (n=35), n (%)	Oman (n=33), n (%)	Total (N=68), n (%)	Statistics	
					Chi-square value (*df*)	*P* value
Email		32 (91.4)	21 (63.6)	53 (77.9)	9.0 (1)	.003
**Social media**						
	WhatsApp	1 (2.9)	21 (63.6)	22 (32.4)	18.6 (1)	<.001
	Twitter	0 (0.0)	7 (21.2)	7 (10.3)	6.3 (1)	.008
	Facebook	1 (2.9)	5 (15.2)	6 (8.8)	2.2 (1)	.10
	Instagram	0 (0.0)	2 (6.1)	2 (2.9)	1.7 (1)	.19
	Messenger	0 (0.0)	2 (6.1)	2 (2.9)	1.7 (1)	.19
	Other	1 (2.9)	0 (0.0)	1 (1.5)	1.5 (1)	.32

### Attitudes Toward Patients Bringing Material From the Internet

In addition to surgeons’ behaviors, we wanted to know about their attitudes toward e-patient behaviors, particularly regarding the patient-doctor relationship. Table 8 shows results from statements beginning with “If a patient brought some health-related information to a consultation...” and based on a Likert scale, ranging from 1 (strongly disagree) to 7 (strongly agree).

The surgeons from both the countries felt more positive than negative about patients’ bringing health-related information from the internet to the consultation, and there was no difference between the 2 countries on this question. There was an inverse effect with age for Germany, but there was no other effect by age (*P=*.11), gender (*P=*.69), or condition type (*P=*.52). There was no effect between the answers to this question and the number of electronic interactions with patients via email or the percentage of patients with whom they communicate via email (*P=*.14).

The surgeons from both the countries were prepared to correct wrong or incomplete information, and there was no difference between the 2 countries on this question and no associations with age (*P=*.69), gender (*P=*.95), or condition type (*P=*.95).

Although the surgeons from neither country felt very strongly about the loss of control, there was a difference between the 2 countries on this question, with the German surgeons feeling more strongly about this. There were no associations by condition type (*P=*.71) but an overall association by gender, with males feeling more strongly about this issue (*P=*.005).

The German surgeons felt more strongly than the Omani surgeons about time-consuming consultations, and there was an inverse association with age among the Omani doctors (*r*=-0.282; *P=*.003).

Generally, the surgeons felt that the resultant communication would lead to an improvement in the patient-doctor relationship, and there was no difference between the 2 countries on this question and no effect by age (*P=*.99), gender (*P=*.52), or condition type (*P=*.97).

The surgeons were disinclined to prescribe different medications, and there was no difference between the 2 countries on this question and no effect by age (*P=*.34), gender (*P=*.06), or condition type (*P=*.83).

Finally, because surgeons’ attitudes can be associated with the amount of interaction with e-patients, we looked for any associations between the most common activities and the answers to the above 6 questions in general and on the country level. Of all these, the only association was between the percentage of patients bringing in information from the internet (Pearson *r*=0.278; *P*=.004) and the likelihood that the surgeons would prescribe a desired medication.

**Table 8:** Summary of responses to attitude statements.

Item	Germany (n=49), mean (SD)	Oman (n=59), mean (SD)	Overall (n=108), mean (SD)	Statistics	
				*t* value (*df*)	*P* value
I think it is generally positive.	4.20 (1.5)	4.03 (1.7)	4.11 (1.6)	−0.56 (105)	.58
I am prepared to correct wrong, incomplete, and misunderstood information.	5.12 (1.7)	5.03 (1.3)	5.10 (1.8)	−0.11 (104)	.91
I sometimes feel I might lose authority and control.	3.43 (1.9)	2.32 (1.3)	2.82 (1.7)	−3.53 (106)	.001
I expect a more time-consuming patient visit than with uninformed patients.	5.10 (1.4)	3.92 (1.6)	4.45 (1.7)	−4.03 (106)	<.001
The physician-patient relationship will be improved by better communication.	4.41 (2.0)	4.81 (1.8)	4.63 (1.9)	1.09 (105)	.28
I would be more likely to prescribe a desired medication than if the patients were uninformed.	3.08 (1.7)	3.53 (1.9)	3.32 (1.8)	1.27 (105)	.21

## Discussion

### Principal Findings

This paper reported the e-patient–related activities and attitudes of surgeons at 2 sites in Germany and Oman. There are many similarities between the 2 groups; indeed, many of these similarities can be found in other studies. There are, however, differences between the 2 sites, particularly in the methods of communication between the surgeons and patients. The discussion below will explore some of these similarities and differences in light of the literature.

As we based a large portion of our questionnaire on the one produced by Moick and Terlutter [27] (who studied general practitioners, orthopedists, and dermatologists in Germany), we shall refer frequently to their paper as a point of comparison, although the other literature will also have a bearing on our results.

### Internet Usage

The surgeons in this study generally used the internet far more than the doctors in Moick and Terlutter’s study [27], and the percentage of work-related time on the internet was also higher (52.0% as opposed to 25.3%). Given that the work of Moick and Terlutter [27] was published in 2012 and earlier studies also generally show lower figures of usage [13], this difference may be a reflection of the fact that overall internet usage is growing around the world.

Overall, the German surgeons spent a greater percentage of their online time on work-related activities than the Omani surgeons. Earlier studies of internet usage by doctors [13] showed differences associated with gender, but even those figures were not always consistent. Our study also shows few differences between males and female activities.

### Surgeons’ Knowledge, Use, and Recommendation of Sites and Apps

As was noted in the Introduction section, patient engagement has led to better health outcomes [20-22], and so it is important to consider the level of engagement afforded by the surgeons with their patients. Several studies have found that doctors recommend sites to patients, and this has been happening for many years [13], and the surgeons in this study confirm this practice.

With the surgeons in this study, we wished to have a more detailed knowledge of their baseline knowledge and usage of various sites and apps. Although the aims of the study did not require that we conduct detailed statistical analyses on the various sites and apps, we were able to gather a greater sense that, overall, the surgeons’ knowledge and use of the internet were extensive. This provided important contextual information so that we could be surer that any lack of site recommendation for patients would not be simply because of a lack of knowledge or awareness of these sites.

In this light, among the Omani surgeons, the high number of Google recommendations (11/29, 38%) is of great concern. Unsurprisingly, patients have long been using these general search engines as a starting point to health sites [11], and there is a need for patients to have more guided searches. For some time, it has been suggested that surgeons need to be taught about the use of the internet, including useful patient education sites and site evaluation [4,10,28], in a similar way that they are taught other skills and knowledge. It is not merely knowledge of these sites (as these surgeons have indicated that they have this knowledge), but rather transferring that knowledge into useful guiding information for their patients. This should be addressed in the Omani surgeons’ education.

### Communication With the E-Patient

From our perspective, among the most interesting results were the figures on electronic communication with patients. For several years, studies have shown that, in spite of some reservations, many physicians are satisfied with email communication with patients and have continued to use email as a standard method of communication [13]. (Most studies have shown, however, that the actual percentage of patients whom doctors email is low [13].) In our study, given that the German surgeons used the internet for work-related activities more than the Omani surgeons, the German surgeon’s higher electronic communication figures (71% vs 56%) were not surprising.

Noteworthy in this study, however, was the great difference in the *methods* of communication between the 2 countries. Of the surgeons who communicated electronically with their patients, the German surgeons almost used only email (91%), whereas social media usage for patient communication was virtually zero. The Omani figures are very different: although 64% of the Omani doctors used email, an equal number used WhatsApp, and overall, social media tools were used far more than standard email.

It would be dangerous to speculate too deeply on the Omani figures, and this could be the subject of later research. There are, however, 2 possible reasons based on sociological and recent historical differences between the 2 countries:

1. In general, Omani society is very close knit, and it is not unusual for doctors and patients to be related. Approximately 52% of Omani marriages are consanguineous, and more than 75% of these marriages are with first cousins [29]. Relatives are frequently recognized by common surnames related to tribes and region of origin. Within these social structures, sharing of private telephone numbers is reasonably common. As WhatsApp is based on telephone numbers and many Omani doctors use the same phone for professional and personal reasons, their patients will already have access to their numbers and their WhatsApp accounts, and it is not uncommon for patients to contact doctors on more than one social media platform simultaneously. It should be noted, moreover, that of the Omani surgeons in the department, 47% (39/83) surgeons are Omani nationals, so the impact of this social characteristic is uncertain. This would be an area to be explored in more research.
2. Although Oman was not affected by the 2011 Arab Spring to the same extent as many other countries in the region, the power of social media became obvious during that time [30,31]. Since then, social media, especially, WhatsApp and Facebook, have become a part of daily life for people living in the Arab world, including Oman [32,33].

There may be a third reason: an overall growth in patient communication with social media. Currently, although there is international recognition of the role of social media in medical practice [5,34], its usage is frequently in the form of informal patient communities, just as the Web was used for patient communities before social media. It is possible that the sociological and historical elements outlined above have merely provided an early impetus—indicating a coming change—and the fact that just as email communication by doctors was very low barely 10 to 15 years ago [13] and has increased over time, social media communication between patients and doctors may also increase in the future.

The implications for medical education are important: currently, medical education communication teaching still focuses on face-to-face communication teaching, in spite of the fact that there has long been a call for email communication to be explicitly taught [4]. Just as email communication with patients grew mostly because of demand from patients, social media communication may also do so. There are already calls for social media communication to be included in communication teaching [4], and this study indicates the urgency of that call. Part of this training will have to include careful management, as email is far less intrusive than social media, and the impact on physician’s time (and resultant physician burnout) will be potentially devastating if social media communication is not managed correctly.

Irrespective of the differences between the German and Omani surgeons, both groups show that more than half of the surgeons communicate electronically with their patients, thereby affording the opportunity for Web-based patient engagement; as indicated above, the literature has shown a positive association between patient engagement and better health outcomes [20-22], so we anticipate that this level of interaction will have a positive impact on overall health care delivery.

### E-Patients Bringing Information From the Internet

From our study, the high percentage of surgeons from Germany and Oman reporting patients bringing information to them from the internet is consistent with the literature [13]. The percentage of patients who bring material from the internet remains relatively low, although higher than earlier studies [13], and so, these numbers may increase in the future. In the Introduction section of this paper, reference was made to 65% to 80% of patients who seek information on the internet and do not discuss it with their doctors, primarily because of their doctors’ attitudes toward internet-based information [19,35]. These attitudes are explored in a little more detail below.

Other literature has found an association between recommendations from the doctor and patients bringing information from the internet [14], but we did not find that association in our study. Similarly, previous studies have indicated that when patients with chronic diseases have access to the internet, they have used it for seeking health-related material more than patients with acute conditions [16]. Again, however, this was not reflected in our study. Part of the reason for not finding these trends in our study may be a normalization of e-patient activities. As the percentage of patients searching for information on the internet increases and *e-patient* activities become normal patient activities, recommendations from doctors may have less impact on the numbers of patients searching for material, and patients with acute conditions will search for material as much as patients with chronic conditions. It is to be expected, however, that doctors still have a role in determining *which* sites are visited. Either way, these results emphasize the need to teach doctors how to cope with this phenomenon [28].

### Attitudes Toward Patients Bringing Material From the Internet

The comparison of our results with those of Moick and Terlutter’s study [27] shows many similarities. These surgeons were generally positive about patients bringing material from the internet and were prepared to correct patients’ incorrect information.

Indications from the literature are that patients are increasingly requesting prescriptions by name, having found the information from both advertisements in traditional media and also from social media and other electronic sources [14], and this undoubtedly places pressure on doctors. Similar to Moick and Terlutter’s sample [27], however, these surgeons were somewhat reluctant to change their prescription based on the patients’ findings. Where the surgeons were willing to change prescriptions, this change was positively associated with the percentage of patients bringing information. Whether or not this association is causal and the exact nature of the possible causality would need to be studied further. It may be that the sheer number of requests leads to changes or it may be that surgeons who are more open to having their patients bringing information are correspondingly more open to changing their prescriptions based on patients’ desires. This is entirely plausible, given that 2 medications may have almost the same impact, and if a patient really does have a preference for a particular medication, and there is no harm to come from it, then changing the prescription could be easily accommodated.

These surgeons envisaged an improvement in the patient-doctor relationship caused by the better-informed patient. This is a controversial discussion point in the literature, in which some studies have found a negative impact, whereas others have found results similar to ours and have seen that the more engaged patient has resulted in improved health care delivery [1,20,21]. Qualitative studies [1] have found that part of the reason for the positive feeling is that physicians can deal with more important and profound issues rather than getting bogged down in trivial explanations.

The differences between the German and Omani figures, however, are also noteworthy. The Omani surgeons were significantly less concerned than the German surgeons and Moick and Terlutter’s sample about the loss of control. Other studies have also indicated that even when physicians are generally positive about well-informed patients, sometimes arguing with patients over irrational points can lead to a fear of lack of trust in the doctor’s ability [1]. Again, this lack of fear among the Omani surgeons may have to do with sociological environment in which these surgeons function, and one may hypothesize that the somewhat relaxed social strata in Oman may lead to less concern about losing control.

The other difference was the fear of loss of time; again, the figures show that the Omani surgeons were far less concerned than the German surgeons and those in Moick and Terlutter’s sample and also in other studies [1]. This, also, may be related to cultural differences, as research indicates that Arab culture can be considered *polychronic*, having a more flexible approach to time and appointments than one may find in the German culture [36-39]. The fact that the Omani surgeons’ fear over loss of time was negatively associated with age may indicate changes in attitudes of the younger generation of doctors in Oman.

### The Larger Context of Medical Education and Participatory Medicine

This paper focuses on the surgeons working with e-patients. As this is already a lengthy paper, it would not be wise to broaden it much further, although it is necessary to look a little at the broader context.

Reference has already been made to the implications of these findings for medical and health education. In short, these findings reinforce and then extend the notion that health education needs to be tailored to meet the demands of the e-patient, specifically on the effective management of electronic communication in health care.

In addition, moreover, there is the entire field of participatory medicine, in which patients move away from *compliance* to active participation in their health care [40], further trending also toward a *role convergence* between the patient and doctor [2]. But the changes are not occurring with patients only, as doctors are also changing. Many previous studies on physicians’ use of technologies showed variation within the demographic indicators of age and gender, although even then there were indications that the differences were not always clear-cut [13]. In this study, the differences are further blurred, and other factors, such as culture or historical events, appear to have a greater impact on the differences. We would argue that this is to be expected as these technologies become mainstream, and are no longer used only by early adopters. Indeed, reflecting on these results, the literature shows a reduction in earlier reports of doctors lagging behind patients in embracing the internet to a mirroring of e-patient and the rise and development of the *empowered physician* (*e-physician*) [41] who will perform these tasks as a normal part of their work.

The activities and especially the positive attitudes of the surgeons in this study appear to show a great opportunity for increased patient participation and engagement in their health care, and this bodes well for future positive health care delivery. Previous studies have indicated that doctors’ attitudes strongly impact patient activities, but this has not been strongly supported in this study. It may be that e-patients are now less impacted by doctors’ attitudes than before or it may be that there is a tipping point where a change from negative to positive is the main difference, after which the patients take greater control irrespective of small differences in surgeons’ positive attitudes.

### Limitations of the Study

Although the study was conducted in 2 countries, the centers were localized to the 2 hospitals associated with the researchers’ universities. That said, the figures for Germany conform to many of the trends found elsewhere, and Oman is a relatively small country with few surgical centers and only 2 medical schools. A second limitation is the sample size of a total of 108 surgeons.

### Conclusions

Recognizing that doctors’ electronic interactions with patients impact health care delivery, this study has examined German and Omani surgeons’ interactions with and attitudes toward e-patients. We have seen that, overall, these doctors are comfortable with many e-patient activities and that the doctors’ attitudes do not have a significant impact on the e-patient activities. This may be because the e-patients see these activities as part of their normal lives and are performed irrespective of doctors’ opinions about the activities. Either way, the approach by these surgeons has created the opportunity for greater patient participation and engagement, and the literature indicates that this opportunity should have a great positive impact on health care delivery.

In addition, however, there were differences between the 2 countries, most notably in the methods of electronic communication, with the German surgeons using mainly email and the Omani surgeons’ heavy use of social media. Further research needs to be performed to determine the extent to which this difference results from cultural differences and recent historical events; it is also possible that the difference is an early indication of e-patients wishing to shift communication from email to social media.

Beyond medical practice, there are implications for medical education, and medical schools need to ensure that medical students receive comprehensive training on working with the e-patient, including appropriate electronic communication with patients.

## Appendix

Multimedia Appendix 1 Checklist for Reporting Results of Internet E-Surveys checklist and corresponding actions taken in the paper.

Multimedia Appendix 2 Questions for physician questionnaire.

Multimedia Appendix 3 More detailed charts.

## Abbreviations

CPD: Continuing Professional Development
