# Supplementary material for: Surgeons’ Interactions With and Attitudes Toward E-Patients: Questionnaire Study in Germany and Oman
Source: J Med Internet Res. 2020 Mar 9;22(3):e14646. doi: 10.2196/14646 (PMC7091032; doi:10.2196/14646)
Supplement: Multimedia Appendix 1 [file jmir_v22i3e14646_app1.docx]

Surgeons’ interactions with and attitudes towards e-patients: a questionnaire study in Germany and Oman

Appendix 1: CHERRIES Checklist and corresponding actions taken in the paper

The Table reproduces the CHERRIES checklist from [1], Table 1. The first two columns are from the original Table 1, and the third column is a listing of the location in the paper where each item has been provided, with an explanation, if required. In most cases, the explanation is a simple copy-and-paste quotation (indicated in quotation marks) from the paper.

Table 1: Checklist for Reporting Results of Internet E-Surveys (CHERRIES)

| 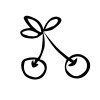 | **Checklist for Reporting Results of Internet E-Surveys (CHERRIES)** | |
| --- | --- | --- |
| ***Item Category*** | ***Checklist Item*** | **If each item is addressed in the paper, the location in the paper where each item has been provided / explained / listed.** |
| **Design** |  |  |
|  | Describe survey design | In **Methods, Questionnaire**:  “In Germany, the questionnaire was delivered to all surgeons from General, Transplant, Visceral, Heart and Orthopaedic & Trauma Surgery from the University Hospital, Tübingen and the affiliated *Berufsgenossenschaftliche Unfallklinik* (BGU), Tübingen. The questionnaire was on paper and in electronic format, using Google Forms. English fluency could be assumed among German doctors working in an academic environment, so the questionnaire was administered in English.”  “In Oman, the questionnaire was delivered to surgeons in the Department of Surgery at Sultan Qaboos University Hospital (SQUH) (including those surgeons affiliated to the Department from the Oman Medical Association). The questionnaire was on paper and in electronic format, using Survey Monkey. Because all doctors in Oman need to be fluent in English, the questionnaire was delivered in English.” |
| **IRB (Institutional Review Board) approval and informed consent process** |  |  |
|  | IRB approval | In **Methods, Questionnaire:**  German study:  “Ethics approval was obtained from the University of Tübingen’s Medical Ethics Committee (No. 001/2018BO2).”  Oman study:  “Ethics approval was obtained from the Sultan Qaboos University College of Medicine Research Ethics Committee (MREC#1628).” |
|  | Informed consent | In **Methods, Questionnaire**:  “In all cases, the information sheet and consent form contained the title and brief description of the research project, names and contact details of the researchers, a brief statement about risks to the participants, confidentiality, storage of information (256-bit encryption), the voluntary nature of the participation, and permission to retain (or obtain) a copy of the informed consent form. All surgeons signed the informed consent form or checked an appropriate box on the electronic form. After collection of the paper forms, the signed consent form was separated from the questionnaire, and housed in a separate location.” |
|  | Data protection | *See section on Informed consent above* |
| **Development and pre-testing** |  |  |
|  | Development and testing | In **Methods, Questionnaire**:  “For the core of the questionnaire design, we elected to use the relevant part of the survey form designed by Moick and Terlutter. Moick and Terlutter’s questionnaire was based on issues raised in the medical literature, and they determined it to be internally consistent. The questionnaire consists of six items about attitudes of online informed patients ranging from 1 (“absolutely disagree”) to 7 (“absolutely agree). As a double-check, we inspected the literature that Moick and Terlutter had cited in the construction of their questionnaire. We did this to ensure that those sources did support the construction of their questions, and accepted the questions as valid.  In addition to the questions from Moick and Terlutter, other literature and surgeons were consulted to add further questions. The final version of the questionnaire is to be found in Appendix 2.” |
| **Recruitment process and description of the sample having access to the questionnaire** |  |  |
|  | Open survey versus closed survey | In **Methods, Questionnaire Delivery**:  As the surgeons were “directed to the online forms through Uniform Resource Locators (URLs)”, survey was open only to those who had access to the URLs. |
|  | Contact mode | In **Methods, Questionnaire Delivery**:  “The surgeons were contacted through internal electronic mailing lists and WhatsApp groups, and directed to the online forms through Uniform Resource Locators (URLs).” |
|  | Advertising the survey | See *Contact Mode* above. No further advertising was conducted. |
| **Survey administration** |  |  |
|  | Web/E-mail | In **Methods, Questionnaire**:  In Germany:  “The questionnaire was on paper and in electronic format, using Google Forms.”  In Oman:  “The questionnaire was on paper and in electronic format, using Survey Monkey.” |
|  | Context | In **Methods, Questionnaire**:  As only SurveyMonkey and Google Forms were used, there was no webpage information to consider. |
|  | Mandatory/voluntary | This was not on a webpage, but was voluntary based on the email/WhatsApp notices that was sent out. |
|  | Incentives | In **Methods, Questionnaire Delivery**:  “No incentives were offered to the surgeons for the completion of the form.” |
|  | Time/Date | In **Methods, Questionnaire Delivery**:  “The data collection was performed during March – July 2018.” |
|  | Randomization of items or questionnaires | No randomisation was performed, and so this is not mentioned in the paper, |
|  | Adaptive questioning | No adaptive questioning was performed, and so this is not mentioned in the paper. |
|  | Number of Items | In **Methods, Questionnaire**:  Because a strict number of items can be misleading, the survey form is included as Appendix 2 to the paper. “The final version of the questionnaire is to be found in Appendix 2.” |
|  | Number of screens (pages) | In **Methods, Questionnaire**:  “The online version was delivered on a single, scrollable screen, so that the surgeons could review all their answers before submission.” |
|  | Completeness check | In **Methods, Questionnaire**:  “the voluntary nature of the participation”  Because completing the form was voluntary, all questions were also voluntarily completed. |
|  | Review step | In **Methods, Questionnaire**  “The online version was delivered on a single, scrollable screen, so that the surgeons could review all their answers before submission.” |
| **Response rates** |  |  |
|  | Unique site visitor | See **Methods, Questionnaire Delivery**.  “In order to maintain confidentiality, and on the grounds that these are extremely easy to circumvent, no checks or preventative measures through cookies or Internet Protocol (IP) address identification were taken.” |
|  | View rate (Ratio of unique survey visitors/unique site visitors) | See comment above. |
|  | Participation rate (Ratio of unique visitors who agreed to participate/unique first survey page visitors) | See comment above. |
|  | Completion rate (Ratio of users who finished the survey/users who agreed to participate) | See comment above. |
| **Preventing multiple entries from the same individual** |  |  |
|  | Cookies used | See comment above. |
|  | IP check | See comment above. |
|  | Log file analysis | See comment above. |
|  | Registration | As the survey was open, no registration was required. |
| **Analysis** |  |  |
|  | Handling of incomplete questionnaires | In **Methods, Data Analysis**.  All questionnaires were analysed. |
|  | Questionnaires submitted with an atypical timestamp | No timestamping was performed. |
|  | Statistical correction | No weighting was performed |

# References

1. Eysenbach G. Improving the Quality of Web Surveys: The Checklist for Reporting Results of Internet E-Surveys (CHERRIES). J Med Internet Res 2004;6(3):e34. PMID:15471760
