# Supplementary material for: Surgeons’ Interactions With and Attitudes Toward E-Patients: Questionnaire Study in Germany and Oman
Source: J Med Internet Res. 2020 Mar 9;22(3):e14646. doi: 10.2196/14646 (PMC7091032; doi:10.2196/14646)
Supplement: Multimedia Appendix 2 [file jmir_v22i3e14646_app2.docx]

**Questions for physician questionnaire**

1. My Age is ____________ 2. My Gender is female male

3. How many hours a day do you use the Internet?

| - 0 | - 1-2 | - 3-4 | - 5-6 | - 7-8 | - 9-10 | - >10 |  |  |  |  |  |
| --- | --- | --- | --- | --- | --- | --- | --- | --- | --- | --- | --- |

4. What percentage of this time do you use it on work-related activities? Please mark

the appropriate number (in %) by circling the number.

**100%**

**0% 5 10 15 20 25 30 35 40 45 50 55 60 65 70 75 80 85 90 95**

|  |  |  |  |  |  |  |  |  |  |  |  |  |  |  |  |  |  |  |  |  |
| --- | --- | --- | --- | --- | --- | --- | --- | --- | --- | --- | --- | --- | --- | --- | --- | --- | --- | --- | --- | --- |

5. I **know of** this medical/health web site type:

| - Online books (e.g. Google books, atlases, Thieme online) |
| --- |
| - Online videos (e.g. Websurg, DGHC Mediathek |
| - General references (e.g. Up to date, Flexicon, Amboss, Guidelines, WebMD, Medscape) |
| - Networking sites (e.g. Coliquio, Esanum) |
| - Official/ institutional bodies (e.g. Medical or Professional Association, DKFZ) |
| - Online databases (e.g. PubMed, Google Scholar, Web of Science) |
| - Journals (e.g. The Lancet, JAMA, Annals of Surgery, British journal of surgery) - Magazines (e.g. Apothekenumschau, Focus Gesundheit, Men’s Health) |

6. I **use** this website type **at least once per month:**

| - Online books (e.g. Google books, atlases, Thieme online) |
| --- |
| - Online videos (e.g. Websurg, DGHC Mediathek |
| - General references (e.g. Up to date, Flexicon, Amboss, Guidelines, WebMD, Medscape) |
| - Networking sites (e.g. Coliquio, Esanum) |
| - Official/ institutional bodies (e.g. Medical or Professional Association, DKFZ) |
| - Online databases (e.g. PubMed, Google Scholar, Web of Science) |
| - Journals (e.g. The Lancet, JAMA, Annals of Surgery, British journal of surgery) - Magazines (e.g. Apothekenumschau, Focus Gesundheit, Men’s Health) |

7. I **know** of this medical/health app type:

| - Monitoring (e.g. BloodPressure, Accurate Heart Rate) |
| --- |
| - Information (e.g. Up to Date, Amboss) |
| - CPD (e.g. MedScape, NEJM, Lancet Picture of the week) |
| - Tools (e.g. Red List, BMI-Calculator, Nephro-Check) |
| - Surgical videos (e.g. Touch Surgery, iLappSurgery) |

8. I **use** this app type **at least once per month:**

| - Monitoring (e.g. BloodPressure, Accurate Heart Rate) |
| --- |
| - Information (e.g. Up to Date, Amboss) |
| - CPD (e.g. MedScape, NEJM, Lancet Picture of the week) |
| - Tools (e.g. Red List, BMI-Calculator, Nephro-Check) |
| - Surgical videos (e.g. Touch Surgery, iLappSurgery) |

9. Have you heard of the term “e-patient” (to refer to a patient who looks up medical information on the Internet)

| - Yes | - No |
| --- | --- |

10. In you role as a doctor, do you interact with patients via email or through social media?

| - Yes | - No |
| --- | --- |
| If yes, which of these |  |
| - Email | - Twitter |
| - Facebook | - Other _____________________ |

10a) How many of your patients interact with you through emails? Please mark

the appropriate number (in %) by circling the number.

**100%**

**0% 5 10 15 20 25 30 35 40 45 50 55 60 65 70 75 80 85 90 95**

|  |  |  |  |  |  |  |  |  |  |  |  |  |  |  |  |  |  |  |  |  |
| --- | --- | --- | --- | --- | --- | --- | --- | --- | --- | --- | --- | --- | --- | --- | --- | --- | --- | --- | --- | --- |

10b) How many of your patients interact with you through social media? Please mark

the appropriate number (in %) by circling the number.

**100%**

**0% 5 10 15 20 25 30 35 40 45 50 55 60 65 70 75 80 85 90 95**

|  |  |  |  |  |  |  |  |  |  |  |  |  |  |  |  |  |  |  |  |  |
| --- | --- | --- | --- | --- | --- | --- | --- | --- | --- | --- | --- | --- | --- | --- | --- | --- | --- | --- | --- | --- |

11. How many of your patients bring material to the consultation or refer to information that they found on the internet when consulting you? Please mark the appropriate number (in %) by circling the number.

**100%**

**0% 5 10 15 20 25 30 35 40 45 50 55 60 65 70 75 80 85 90 95**

|  |  |  |  |  |  |  |  |  |  |  |  |  |  |  |  |  |  |  |  |  |
| --- | --- | --- | --- | --- | --- | --- | --- | --- | --- | --- | --- | --- | --- | --- | --- | --- | --- | --- | --- | --- |

12. On average, in a month, how many times do you recommended a website or app to a patient?

| - 0 | - 1-2 | - 3-4 | - 5-6 | - 7-8 | - 9-10 | - >10 |  |  |  |  |  |
| --- | --- | --- | --- | --- | --- | --- | --- | --- | --- | --- | --- |

13. If more than 0, name one website or app that you have recommended to patients:

14. The majority of the patients you treat suffer from:

| Choice of: | - Chronic conditions | - Acute conditions | - Both in roughly equal amounts |
| --- | --- | --- | --- |

15. Please indicate the degree to which you agree with the statements below. When answering the following questions, please think of typical patients in your surgery. The questions only refer to information they found online beforehand. We are NOT asking about information that patients are bringing from another consultation with a doctor for a second opinion. This is information from the Internet only.

If a patient brought some health-related information to a consultation…..

1. … I think it is generally positive.

| I absolutely disagree | **1** | **2** | **3** | **4** | **5** | **6** | **7** | I absolutely agree |
| --- | --- | --- | --- | --- | --- | --- | --- | --- |

1. … I am prepared to correct wrong, incomplete and misunderstood information.

| I absolutely disagree | **1** | **2** | **3** | **4** | **5** | **6** | **7** | I absolutely agree |
| --- | --- | --- | --- | --- | --- | --- | --- | --- |

1. … I sometimes feel I might lose authority and control

| I absolutely disagree | **1** | **2** | **3** | **4** | **5** | **6** | **7** | I absolutely agree |
| --- | --- | --- | --- | --- | --- | --- | --- | --- |

1. … I expect a more time-consuming patient visit than with uninformed patients

| I absolutely disagree | **1** | **2** | **3** | **4** | **5** | **6** | **7** | I absolutely agree |
| --- | --- | --- | --- | --- | --- | --- | --- | --- |

1. … the physician-patient relationship will be improved by better communication.

| I absolutely disagree | **1** | **2** | **3** | **4** | **5** | **6** | **7** | I absolutely agree |
| --- | --- | --- | --- | --- | --- | --- | --- | --- |

1. … I would be more likely to prescribe a desired medication than if the patients were uninformed.

| I absolutely disagree | **1** | **2** | **3** | **4** | **5** | **6** | **7** | I absolutely agree |
| --- | --- | --- | --- | --- | --- | --- | --- | --- |
