# Supplementary material for: Surgeons’ Interactions With and Attitudes Toward E-Patients: Questionnaire Study in Germany and Oman
Source: J Med Internet Res. 2020 Mar 9;22(3):e14646. doi: 10.2196/14646 (PMC7091032; doi:10.2196/14646)
Supplement: Multimedia Appendix 3 [file jmir_v22i3e14646_app3.docx]

Surgeons’ interactions with and attitudes towards e-patients: a questionnaire study in Germany and Oman

Appendix 3: More detailed charts

These charts are designed to add further detail to the tables in the body of the paper.

Figure 1: Percentage of time spent on Internet is work-related, by country.

Figure 2: Percentage of patients with whom the surgeons communicate via email, by country.

Figure 3: Percentage of patients with whom the surgeons communicate via social media, by country.

Figure 4: Percentage of patients bringing information from the Internet, by country.

Figure 5: Number of times per month a surgeon recommends a website or app to a patient, by country.

Figure 6:… I think it is generally positive. (By country).

Figure 7:…I am prepared to correct wrong, incomplete and misunderstood information. (By country).

Figure 8: … I sometimes feel I might lose authority and control. (By country).

Figure 9: … I expect a more time-consuming patient visit than with uniformed patients. (By country).

Figure 10: …the physician-patient relationship will be improved by better communication. (By country).

Figure 11: …I would be more likely to prescribe a desired medication than if the patients were uninformed. (By country).
